# The Study of Localized Crack-Induced Effects of Nonlinear Vibro-Acoustic Modulation

**DOI:** 10.3390/ma16041653

**Published:** 2023-02-16

**Authors:** Dariusz Broda, Krzysztof Mendrok, Vadim V. Silberschmidt, Lukasz Pieczonka, Wieslaw J. Staszewski

**Affiliations:** 1 Department of Robotics and Mechatronics, AGH University of Science and Technology, Al. A. Mickiewicza 30, 30-059 Kraków, Poland; 2 Wolfson School of Mechanical and Manufacturing Engineering, Loughborough University, Loughborough LE11 3TU, UK

**Keywords:** nonlinear vibro-acoustic modulation, crack–wave interaction, ultrasound, longitudinal vibration, crack localization effect

## Abstract

The nonlinear interaction of longitudinal vibration and ultrasound in beams with cracks is investigated. The central focus is on the localization effect of this interaction, i.e., the locally enhanced nonlinear vibro-acoustic modulation. Both numerical and experimental investigations are undertaken. The finite element (FE) method is used to investigate different crack models, including the bi-linear crack, open crack, and breathing crack. A parametric study is performed considering different crack depths, locations, and boundary conditions in a two-dimensional beam model. The study shows that observed nonlinearities (i.e., nonlinear crack–wave modulations) are particularly strong in the vicinity of the crack, allowing not only for crack localization but also for the separation of the crack-induced nonlinearity from other sources of nonlinearity.

## 1. Introduction

Recent years have shown a growing interest in nonlinear ultrasonic wave propagation for structural damage detection. All theoretical and application studies in this area can be classified into the analysis of classical and non-classical nonlinear effects. The classical effects are mainly related to higher harmonic generation and have been investigated since the mid-1960s [1,2]. This approach is also well-known when used for damage detection [3,4]. Nonlinear wave propagation has also been investigated for decades [5,6,7,8]. The majority of these investigations are limited to the weakly nonlinear dependence of model parameters. The non-classical nonlinear effects related to ultrasonic wave propagation result from various crack–wave interactions that exhibit various phenomena that include sub-harmonic generation, slow dynamics, or vibro-acoustic modulations, as discussed in [9,10,11,12]. All these non-classical non-linear effects have also been used for structural damage detection (e.g., small fatigue cracks in metals or barely visible delaminations in composites).

**Figure 1:**
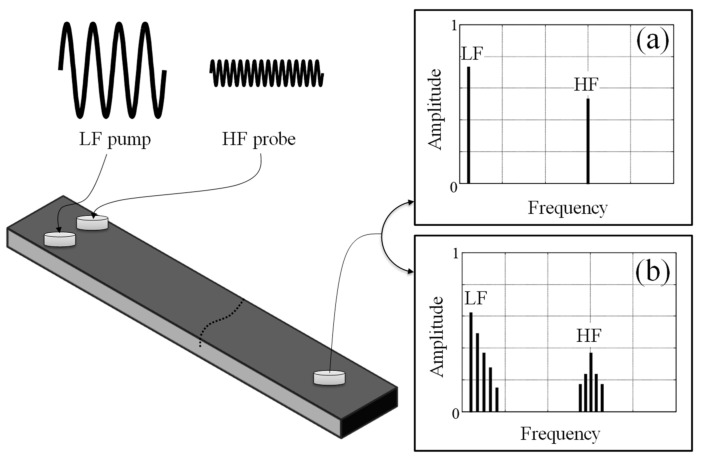
Schematic diagram illustrating the principle of the nonlinear vibro-acoustic modulation technique used for crack detection: (**a**) uncracked structure; (**b**) cracked structure.

The study of the nonlinear vibro-acoustic modulation (VAM) technique [13,14,15,16] has been of particular interest for the last twenty years. The method involves simultaneous ultrasonic and vibration excitation. The weak high-frequency ultrasonic wave (HF) is modulated by the strong low-frequency vibration excitation (LF) in the presence of contact-type material/structural defects (e.g., fatigue cracks in metals or delamination in composites), as illustrated in Figure 1. The nonlinear effects are enhanced in the presence of even low-severity damage, which renders this approach very attractive for Structural Health Monitoring (SHM). Different approaches based on nonlinear vibro-acoustic modulations have been proposed for damage detection, as reviewed in [17]. Previous research investigations in this field utilized various types of transducers, excitation, and signal processing. The method can be used effectively for damage detection and the estimation of damage severity. However, the question of whether nonlinear vibro-acoustic modulations used for damage detection are enhanced in the vicinity of damage—allowing for reliable damage localization—have remained unanswered for a long time. The damage localization problem is also important for reliable monitoring strategy with respect to transducer location. The experimental work in [18,19] demonstrates that damage localization based on nonlinear vibro-acoustic modulations is possible but requires time-consuming laser scanning. A recent numerical study shows that damage localization using VAM is also possible in thick elastic slabs [20].

Although various methods based on nonlinear vibro-acoustic modulations have been developed for structural damage detection, the physical mechanisms of this nonlinear effect are still not fully understood. It appears that this crack–wave interaction is material scale-dependent, strain-dependent, and involves different types of elastic and dissipative effects, as reviewed in [21,22,23]. Some experimental evidence suggests that non-classical nonlinear effects, e.g., modulation transfer due to amplitude-dependent non-hysteretic and non-frictional dissipation, are also involved [24,25]. In addition, this nonlinear effect has been observed not only in macroscopic scales for large strain levels (e.g., closing–opening fatigue cracks—classical nonlinearity) but also in atomic scales for very small strain levels (e.g., anharmonicity of interatomic potential—non-classical intrinsic nonlinearity). The former relates to damage, whereas the latter results from material nonlinearity. Therefore, a physical understanding of the nonlinear mechanisms involved in this non-classical ultrasonic nonlinearity is essential to reliably separate damage-related from material-related nonlinearities. Various modeling attempts have been undertaken to tackle this problem.

Modeling research work related to nonlinear vibro-acoustic wave modulations relates to various crack–wave models. Altogether, these models can be classified into four major groups to simulate nonlinear modulations. These are (1) classical elastic models, (2) hysteretic models, (3) contact models, and non-classical dissipative models (4), as reviewed in [21]. Most reported research work on crack–wave interaction relates to sub- or higher-harmonic generation. Crack modeling in nonlinear vibro-acoustic modulations uses bi-linear stiffness, opening–closing (breathing) motion, clapping mechanisms, and contact acoustic nonlinearity [21,26,27,28,29,30,31,32].

All these models are relatively simple from the physical point of view and involve mainly crack stiffness asymmetry. These models can take the form of an oscillator or relaxator (different force to close and open cracks) and are often used to model adhesive bond defects. Hysteretic models used for nonlinear vibro-acoustic modulations are associated with energy dissipation and involve quasi-static (frequency-independent) and dynamic approaches [33,34]. This group of models also utilize phenomenological approaches, e.g., the Preisach–Mayergoyz (PM) model [33,34,35]. Hysteretic approaches exhibit the dependence of the modulus on the strain-rate sign. The classical contact models are used to simulate the contact of crack faces and often involve adhesion forces. Typical examples in this group include Hertzian and rough-surface contact models used for nonlinear crack–wave modulation modeling [21,36,37]. The non-classical dissipative models are used to explain modulation transfer and are often used in addition to hysteretic models, particularly in mesoscopic materials. The work in this area includes models of solids with inclusions and the viscoelastic model borrowed from polycrystalline materials [21,24]. The latter has been used to explain the acoustic equivalent of the Luxemburg–Gorki effect.

It appears that the majority of these investigations have involved mainly analytical, one-dimensional models or local FE approaches. More recently, the Local Interaction Simulation Approach (LISA) [38] and a non-local approach based on peridynamics [39,40] have also been used for crack–wave interaction modeling. An analytical approach based on phase analysis has been used to model nonlinear crack–wave interactions in [41]. It is important to note that all these crack–wave models have been used mainly to explain nonlinear vibro-acoustic modulations in ultrasonic wave propagation. To our knowledge, no modeling attempt has been made to study the damage localization effect related to nonlinear vibro-acoustic modulations. The current paper aims to address this problem. This is the major novelty of the research undertaken.

The objective of this work is to investigate the localization effect of the nonlinear vibro-acoustic modulation in beams. We attempt to answer the question of whether this effect is localized and enhanced in the vicinity of the crack, allowing for reliable damage localization. The low-frequency vibration excitation is modeled longitudinally, and this is also a challenge since most damage detection applications in this area involve transverse excitation that produces more pronounced modulations. The focus is on numerical simulations. Two crack–wave interaction models—leading to nonlinear vibro-acoustic modulations—are investigated using finite element modeling. Numerical simulations are validated experimentally.

The structure of the paper is as follows. Section 2 briefly explains the nonlinear vibro-acoustic modulation technique used for structural damage detection together with the nonlinear crack–wave models used to explain these modulations. Simulation results are presented in Section 3 and validated experimentally in Section 4. Finally, the paper is concluded in Section 5.

## 2. Numerical Models of Vibro-Acoustic Interactions in Cracked Beams

**Figure 2:**
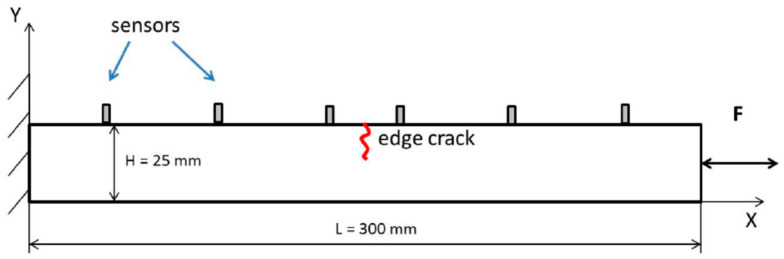
Schematic view of the beam used in FE simulations. Please note that the size and location of the crack, as well as the locations of sensors along the beam, depend on various simulation arrangements used.

This section describes the FE models, developed in *Hexagon MSC Marc* software [42], used to analyze nonlinear crack–wave interactions in beams resulting from combined longitudinal vibration and ultrasonic excitations. The structure under investigation was a cantilever aluminum beam with the size of 300 × 25 × 10 mm, as illustrated in Figure 2.

### 2.1. Crack Models

The beam was meshed with 2 × 2 mm two-dimensional, first-order plane stress finite elements. Five different crack locations were assumed at a distance ranging from 50 mm to 250 mm from the free end of the beam. In all cases, three different depths of the crack were assumed, namely 4 mm, 6 mm, and 8 mm. The 8 mm crack depth corresponded to approximately 1/3 of the beam thickness. Three different crack models were considered.

(1) Bi-linear model of elasticity

**Figure 3:**
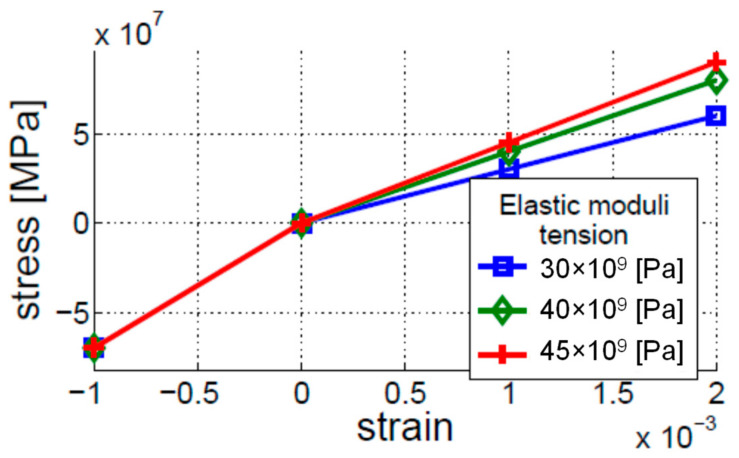
Stress–strain relations applied for the bi-linear model of elasticity for three different crack depths simulated.

This model gives the mathematical function for the elasticity to mimic the closing–opening action of the crack. The elasticity modulus for the compressed cracked beam is the same as for the intact (i.e., uncracked) beam. In contrast, the elasticity modulus for the beam in tension is lower. The bi-linear model provides a good approximation of the breathing crack when beam vibrations are analyzed [21]. For the analysis of vibro-acoustic modulations, however, the stiffness reduction is not as important as the crack opening action. The latter allows for gap creation, which significantly alters the propagating ultrasonic HF wave. The bi-linear model was simulated using three different input levels of nonlinear elastic functions, as shown in Figure 3. The three curves correspond to the three different crack depths under investigation.

(2) Open crack model

The reduced value of Young’s modulus (i.e., reduced from the original value of 70 GPa to the smaller value of 60 GPa) was applied to the twenty-four elements in the middle of the beam to simulate the crack.

(3) Breathing crack model

**Figure 4:**
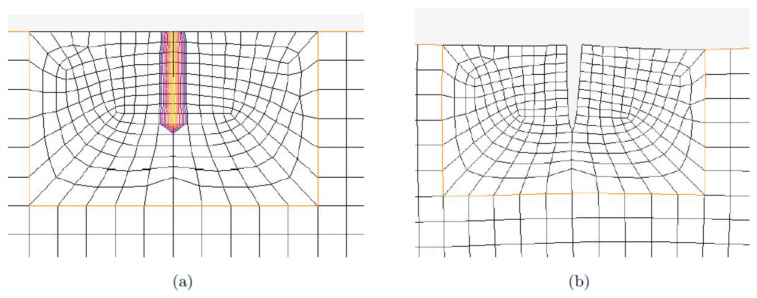
Breathing crack model in *MSC Marc*. The vicinity of the crack meshed for (**a**) closed crack; (**b**) open crack.

The closing–opening action of the crack in this model assumes that the crack is a barrier for the propagating ultrasonic wave, decreasing its amplitude while the beam is in tension. Other phenomena that may contribute to nonlinear modulations were neglected. The contact between crack edges was simulated with the penalty formulation between the contacting bodies. In addition, friction was introduced using the classical isotropic Coulomb friction model. The double-sided, node-to-segment contact was employed. The contact tolerance was set to 2 nm in order to prevent the closing of the crack when crack faces were not touching each other. The nodal stress-based separation detection algorithm was used. This algorithm was selected due to being less sensitive to the element size than the alternative algorithm based on nodal forces. The beam was meshed with the 2D plane-stress elements number *3* (four-noded isoparametric elements with bilinear interpolation) and elements number *201* (three-noded triangular elements with linear interpolation functions). These elements are lower-order elements, which are preferred in contact analysis. The basic element size applied was equal to 2 × 2 mm. Smaller elements were used to mesh the region around the crack (see Figure 4). The implicit, single-step Houbolt dynamic transient operator was employed. A variable step was used in numerical simulations, with a maximum step of 75 µs. The linear interpolation was applied before further processing in MATLAB since the variable time step was employed.

### 2.2. Modal and Ultrasonic Excitation

**Figure 5:**
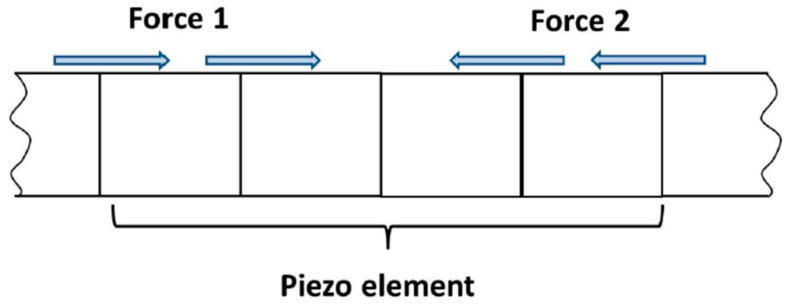
Simplified piezo-model used for the HF ultrasonic excitation.

The vibro-acoustic modulation technique investigated employed low-frequency (LF) vibrations and high-frequency (HF) ultrasonic excitation. The LF excitation was simulated by the application of a harmonic force to a free end of the beam. The HF excitation was simulated initially in the same way. In addition, an alternative model of HF excitation was used. Piezoceramic discs—used in experimental studies (see Section 4) for HF excitation—were modeled numerically. The tension–compression movement of the actuator was simulated using a pair of horizontal forces of reverse direction, applied to four elements at the top of the beam. This simulated an 8-mm-wide piezoceramic actuator. The HF excitation used in this arrangement is illustrated in Figure 5, where the force direction is marked for the action of compression.

The LF excitation in nonlinear acoustics usually corresponds to one of the natural frequencies of the structure investigated. The HF frequency is usually selected arbitrarily. The same approach as in previous investigations [11,12,13,14,15,16,17,18,19] has been undertaken in this study. The LF excitation frequency was equal to 4248 Hz. This value corresponded to the first longitudinal vibration mode of the analyzed beam. The frequency of HF excitation was equal to 100 kHz. Since numerous higher harmonics are generated in the model, both excitation frequencies were selected carefully, so that the frequency of sidebands did not match the frequency of the higher harmonics of the LF excitation.

### 2.3. Vibro-Acoustic Responses and Indicators of Nonlinearity

Ultrasonic responses were analyzed at eight different (transducer) locations along the beam, as shown in Figure 2. Sensor positions were fixed with respect to the location of the crack at 6, 30, 70, and 110 mm to the left and right of the crack. Once the responses were gathered, power spectra were calculated to reveal modulation sidebands around the ultrasonic HF carrier component. The frequency spacing of sidebands corresponded to the frequency of the LF excitation. The level of nonlinearity can be assessed using the amplitude of the modulation sidebands. The amplitude of the first two pairs of sidebands was used, i.e., the first left (*L*1) and the first right (*R*1) sidebands. The level of nonlinearity was also assessed using the modulation intensity coefficient *R*, defined as

(1) $$R=\frac{R1+L1}{2\times T_{HF}}$$

where *T_HF_* is the amplitude of the ultrasonic HF carrier component assessed from the response spectrum; the above parameters were used to study the crack localization effect.

## 3. Numerical Simulation Results

The nonlinear interaction of longitudinal vibration and ultrasound in beams with cracks was investigated using numerical simulation. The work involved three different crack models, described in the previous section, i.e., bi-linear elasticity, open crack model, and breathing crack model. This section describes the results of nonlinear vibro-acoustic modulations for the three crack models investigated.

### 3.1. Bi-Linear Crack Model

**Figure 6:**
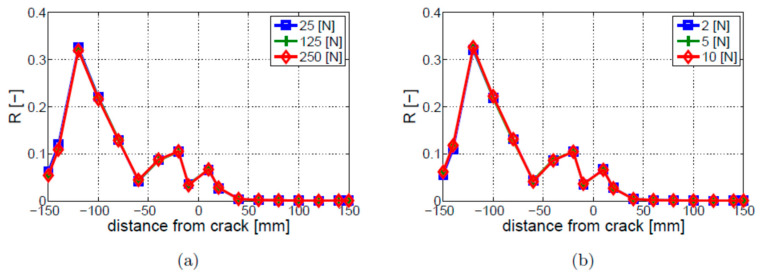
Parametric study of excitation amplitude for the bi-linear crack model and different amplitude levels of (**a**) vibration LF excitation; (**b**) ultrasonic HF excitation.

The bi-linear crack model, described in Section 2.1 as model (1), was investigated. The ultrasonic HF excitation was modeled initially as a harmonic force applied to the free end of the beam. The parametric study of the excitation force amplitude was firstly performed. Three different amplitude levels were used in these investigations for vibration and ultrasonic excitations. Figure 6 gives the values of the *R* parameter for responses measured at different locations indicated on the horizontal axis. The value of 0 mm on this axis corresponds to the crack location, whereas other distance values indicate response measurement locations with respect to the crack, i.e., negative distance values are measurements to the left of the crack and positive distance values are measurements to the right of the crack. The results for the vibration LF excitation are given in Figure 6a, whereas the results for the ultrasonic HF excitation are presented in Figure 6b. The results show that the level of vibro-acoustic modulation nonlinearity does not depend on the amplitude level of LF and HF excitations when the bi-linear crack model is used. This is due to the model used. The bi-linear crack model does not involve any contact of crack asperities, as further explained in Section 3.3.

**Figure 7:**
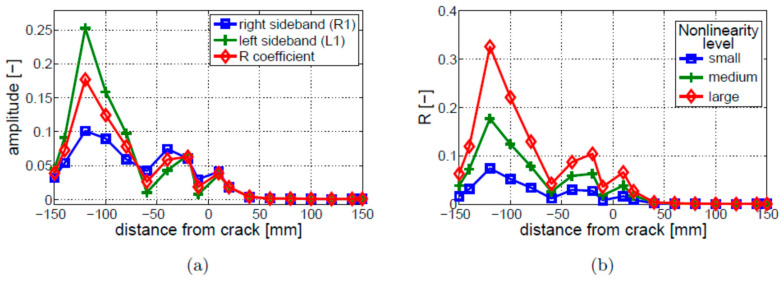
Parametric study of nonlinear vibro-acoustic modulations for the bi-linear crack model: (**a**) modulation intensity; (**b**) crack depth. Small, medium, and large nonlinearity levels correspond to three different crack depths modeled using the stress–strain curves given in Figure 3.

Different nonlinear indicators and levels of nonlinearity were then investigated. The results presented in Figure 7 demonstrate that major differences were observed near the fixed end of the beam. Figure 7a shows that the amplitude level of the first left sideband (*L*1) is significantly larger than the amplitude level of the first right sideband (*R*1). The value of the R coefficient is between the above two amplitude values. Figure 7b gives the values of the R coefficient for different levels of nonlinearity (or crack depth), corresponding to the stress–strain curves presented in Figure 3. The results show that the larger the difference between the elastic moduli in tension and compression, the higher the level of the R coefficient.

**Figure 8:**
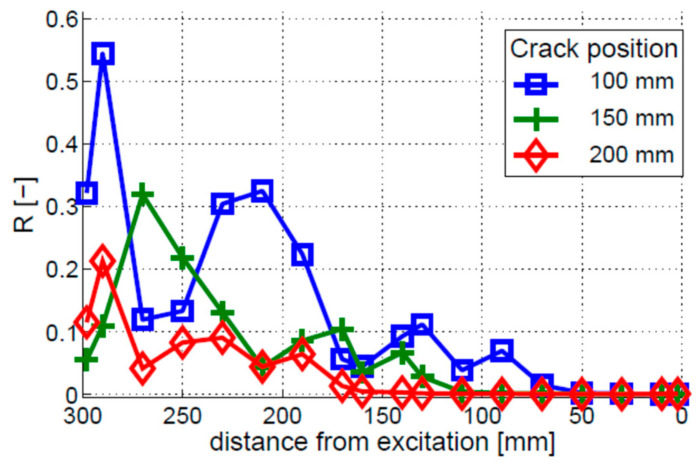
Crack localization effect for the bi-linear crack model. Three different single-crack locations are investigated, i.e., the cracks positioned 100, 150, and 200 mm away from the free end of the beam.

The crack localization effect was then investigated. The results for three different crack positions are given in Figure 8. The coefficient of modulation intensity R starts increasing shortly before the crack and continues to grow until the fixed end of the beam. However, this growth is not monotonic; local peaks can be observed in the R curves, making crack localization difficult, if not impossible.

**Figure 9:**
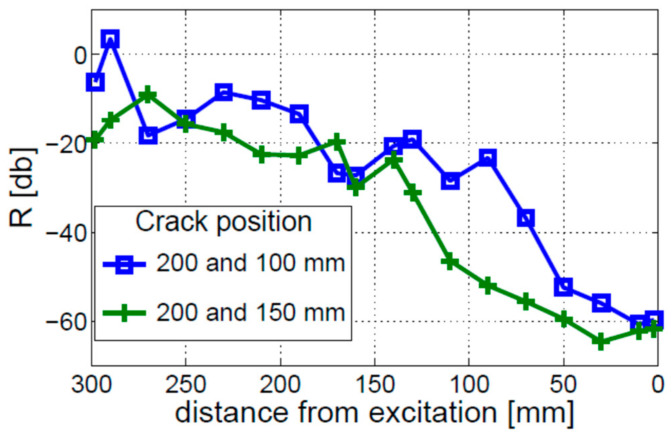
Crack localization effect for two different double-crack scenarios. Numerical simulations performed for the bi-linear crack model.

In the next step, the nonlinear behavior of the beam with two cracks was investigated. Two different double-crack scenarios were considered: (1) cracks located 100 mm and 200 mm away from the free end of the beam; (2) cracks located 200 mm and 150 mm away from the free end of the beam. The results for both double-crack scenarios are presented in Figure 9 for different response measurements taken with respect to the point of excitation. The observed distribution of nonlinearity for both scenarios is similar to the single-crack scenario, described above. The coefficient of modulation intensity R starts to increase near the crack positioned closer to the free end of the beam and stays relatively large till the fixed end of the beam. A very small local increase in nonlinearity can be seen only for the 200 mm and 100 mm crack positions. In the second double-crack scenario, i.e., when the cracks are relatively close to each other (200 and 150 mm), the presence of the second crack is not visible in the local distribution of nonlinearity. Again, the localization effect of nonlinearity for the double-crack scenario would be very difficult to use for crack localization. The third possible double-crack scenario (i.e., cracks located 100 mm and 150 mm away from the free end of the beam) was not investigated as it would be another example of closely spaced cracks. Given that the two investigated cases yielded negative results for crack localization, the third result, even if positive, would not change the entire outcome of unreliable crack detection.

**Figure 10:**
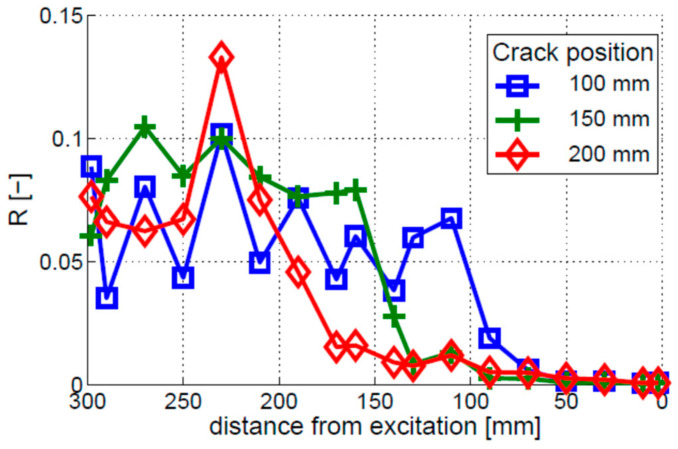
Crack localization effect for three different crack positions. The bi-linear crack model was used. The simplified piezo model was applied for the ultrasonic HF excitation.

The simplified piezo model, described in Section 2.3, was used in the next step for the ultrasonic HF excitation. Figure 10 presents the numerical simulation results for the three different crack positions that were considered. The observed distribution of the coefficient of modulation intensity R is similar to the previously presented model with the harmonic force ultrasonic HF excitation. The modulation intensity starts increasing in the vicinity of the crack and stays relatively high till the fixed end of the beam. However, this time, the rate of increase is higher, enhancing the crack localization effect, but only for the crack located 200 mm away from the free end of the beam.

In summary, the bi-linear crack model can be used to analyze the nonlinear vibro-acoustic modulations. However, since this crack model does not form a natural barrier for a propagating ultrasonic wave, the growth in modulation intensity is generally not localized well near damage. The values of the coefficient of modulation intensity R remain large between the crack and the fixed end of the beam. The results also show that the ultrasonic HF excitation modeling approach significantly influences the distribution of nonlinearity along the beam.

### 3.2. Open Crack Model

**Figure 11:**
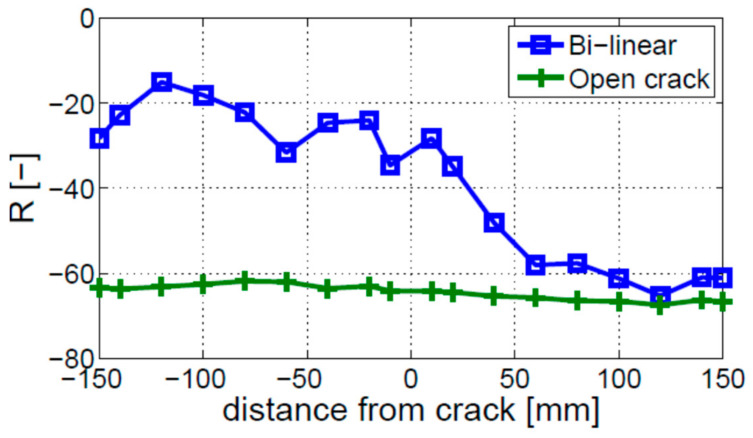
Distribution of modulation intensity coefficient R along the beam for the open crack model used in numerical simulation. The results are compared with the bi-linear crack model.

The open crack model—described in Section 2.2 as model (2)—was investigated to study nonlinear vibro-acoustic modulations. The open crack model results are compared in Figure 11 with the bi-linear model results investigated in the previous section. This clearly shows that nonlinear sidebands are not generated by the open crack model. As a result, the value of modulation intensity R is very low and stays nearly constant along the beam. In conclusion, this model cannot be used for the analysis of the crack localization effect.

### 3.3. Breathing Crack Model

Finally, the breathing crack model described in Section 2.2 as model (3) was used in numerical simulations. Since this model involves the crack opening–closing action, the crack itself is a barrier for the propagating ultrasonic wave. As a result, when the beam is in compression, the wave can easily pass the crack, and when the beam is in tension, the amplitude of the wave is reduced. The ultrasonic HF excitation was initially modeled by the application of a harmonic force to a free end of the beam.

**Figure 12:**
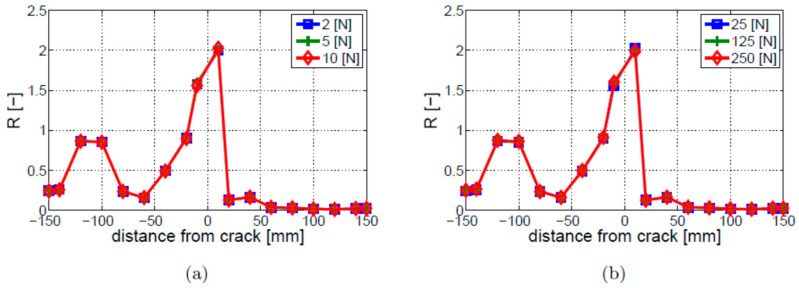
Parametric study of excitation amplitude for the breathing crack model and different amplitude levels of (**a**) ultrasonic HF excitation; (**b**) vibration LF excitation.

Firstly, the effect of the excitation amplitude on the intensity of signal modulation was investigated. The results, presented in Figure 12, show that the modulation intensity coefficient R does not depend on the amplitude of the LF vibration and HF ultrasonic excitations. It is important to remember that overlapping nodes in the model of the crack form a barrier even for small excitation amplitudes, and the model does not take into account the roughness of crack faces. In this model, the ultrasonic wave cannot cross the crack, even for the highest possible separation stresses, which would lead to a closed crack scenario. This is why the nonlinearity does not depend on the excitation amplitudes. Real fatigue cracks would always involve some contact of the internal asperities, even for high opening stresses. The number of asperities in contact would depend on the level of stress; hence, the intensity of nonlinear modulation will depend on the excitation amplitude. The breathing crack model considered here is not able to reflect this dependency.

**Figure 13:**
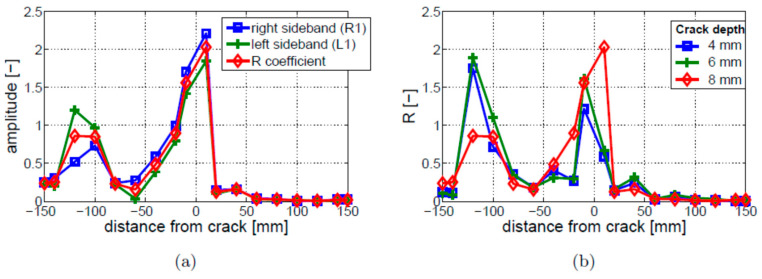
Parametric study of nonlinear vibro-acoustic modulations for the breathing crack model: (**a**) modulation intensity; (**b**) crack depth.

The next step involved the analysis of nonlinear vibro-acoustic modulations. The R, R1, and L1 coefficients, described in Section 2.3, were used in this analysis as indicators of nonlinearity. Figure 13a shows that, although the values of the R parameter are between the values of the R1 and L1 parameters, all three coefficients exhibit values that are closer to each other, if compared with the bi-linear model. The major differences were found near the fixed end of the beam, where the amplitude of L1 (first left sideband) is significantly higher than that of R1 (first right sideband). The results of the crack depth study are presented in Figure 13b. The analyzed parameters exhibit peak values of the R coefficient near the crack (i.e., for the values of distance from the crack close to 0 mm) for all analyzed crack depths. The results also show that the deeper the crack, the larger the value of R at this point. Interestingly, the second local growth in nonlinearity can be observed near the fixed end of the beam (i.e., for the values of distance from the crack close to −120 mm). The level of nonlinearity at this point is not related to the crack depth, and smaller crack depths (i.e., 4 and 6 mm) exhibit larger values of R than in the crack vicinity.

**Figure 14:**
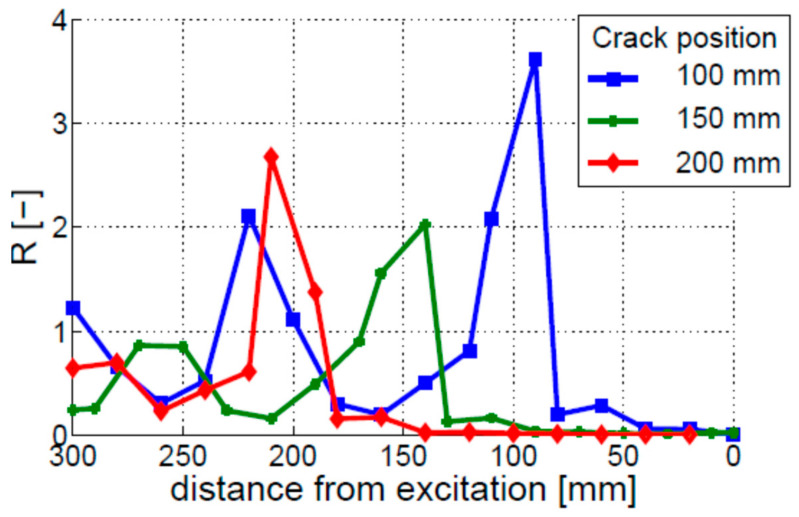
Crack localization effect for the breathing crack model. Three different single-crack locations are investigated, i.e., the cracks positioned 100, 150, and 200 mm away from the free end of the beam.

The crack localization effect was analyzed for three different crack locations, i.e., 100, 150, and 200 mm. The results, given in Figure 14, show that crack locations can be identified by the peak values of R, so the crack localization effect can be observed. However, the 100 and 150 mm cracks exhibit additional peaks in the R characteristics. The locations of these peaks do not correspond to the locations of the crack. Their amplitude is lower than the amplitude of the peaks corresponding to the crack locations.

**Figure 15:**
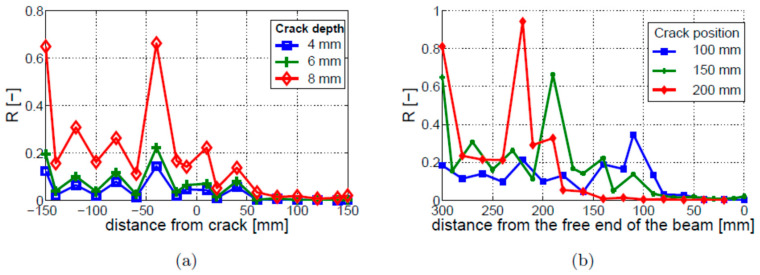
Parametric study of nonlinear vibro-acoustic modulations for the breathing crack model and the piezo model of ultrasonic HF excitation: (**a**) crack depth; (**b**) crack localization effect.

The piezo model of the ultrasonic HF excitation, described in Section 2.2, was also used in the simulated work that involved the breathing crack model. Again, the observed level of nonlinear modulation intensity was found not to depend on the excitation amplitude. Numerical simulation results, shown in Figure 15a, imply that the nonlinearity level is dependent on the crack depth, but this relation is not linear. The modulation intensity is much lower for the 4 mm and 6 mm cracks, when compared to the 8 mm crack. The crack localization effect can be observed in the distribution of the modulation intensity along the beam, as shown in Figure 15b. The modulation intensity is highest when the crack is near the fixed end of the beam and decreases when the crack position moves towards the free end of the beam. The other difference, when compared to the longitudinal HF excitation model, is that only single local peaks—indicating strong nonlinearities—can be found in the R characteristics. However, the highest values of modulation intensity coefficient R are shifted towards the fixed end of the beam with respect to the real crack positions.

### 3.4. Summary of Numerical Investigations

The numerical simulation of nonlinear vibro-acoustic modulation led to interesting results that can be summarized using the following points:

- The open crack model is not able to describe the nonlinear vibro-acoustic modulations, as expected, and cannot be used for the analysis of the crack localization effect.
- The proportionality between the input excitation amplitude and modulation intensity level was not observed in the numerically simulated results that involved the bi-linear and breathing crack models.
- The amplitudes of the first left and right sidebands were found to be similar in the breathing crack model and different in the bi-linear model.
- The proportionality between the crack depth and the modulation intensity level was observed for the bi-linear crack model. This relation is more complex in the breathing crack model and strongly depends on the ultrasonic HF excitation modeling approach.
- The crack localization effect was found in the bi-linear and breathing crack models, although the distribution of nonlinearity along the beam is significantly different for both crack models. The distribution of nonlinearity is also affected by the ultrasonic HF excitation modeling approach.

## 4. Experimental Validation

A series of experiments were carried out to validate the numerical simulation results presented in the previous section. The nonlinear behavior of cracked beams was investigated for different crack depths and positions. Nonlinear vibro-acoustic responses were captured along the beam to analyze the crack localization effect. The work involved different boundary conditions and repeatability tests. This section describes the experimental results.

### 4.1. Experimental Setup and Test Procedure

**Figure 16:**
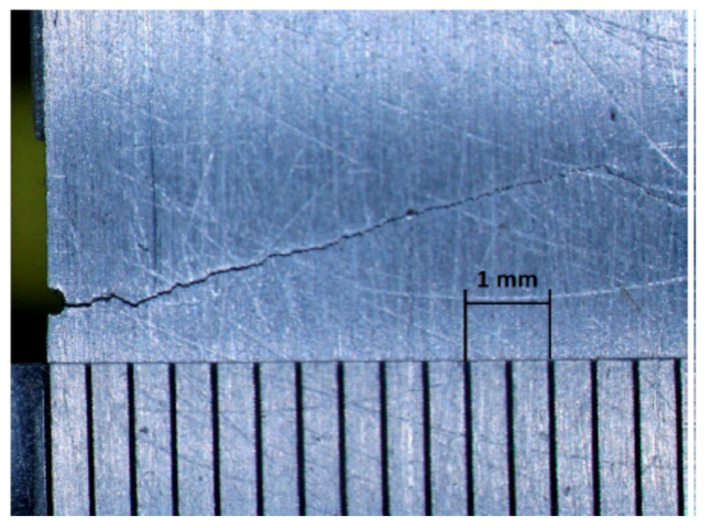
Fatigue crack close-up in one of the aluminum beam components.

The test samples in all experimental investigations were composed of aluminum grade 6082T6 and had the size of 300 × 20 × 10 mm. To initiate cracks in the desired locations during fatigue bending, wire-cut electric discharge machining (EMD) was used to introduce small notches in the beams. Then, three-point bending fatigue tests were performed to initiate and propagate fatigue cracks in beam specimens to desired depths. Figure 16 shows a close-up example of the fatigue crack in one of the samples.

Vibro-acoustic tests were performed using cracked beams to gather nonlinear responses. The beams were analyzed in the free–free and fixed–free boundary conditions. In the free–free boundary conditions, the beams were suspended on two elastic cords, while in the fixed–free boundary conditions, one end of the beam was clamped in a heavy pneumatic vice at the length of 50 mm. A constant clamping force was maintained in all experimental tests.

Two different crack locations positioned at 100 mm and 150 mm from the free end of the beam were investigated. The second location corresponds to the mid-span position of the crack in the beam. Two different crack depths, i.e., 4 and 10 mm, were investigated for specimens with the first (100 mm) location of the crack. The specimens with the second crack location (150 mm) were tested only with the 10 mm crack depth. Thus, altogether, three different vibro-acoustic tests were performed, as summarized in Table 1.

**Figure 17:**
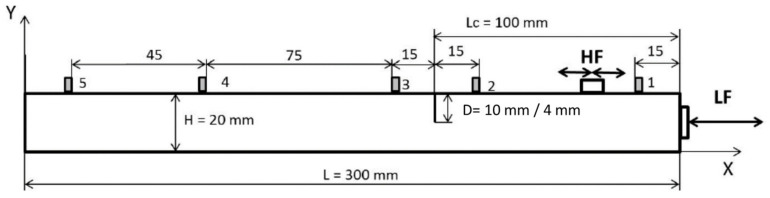
Schematic diagram indicating crack, actuator, and sensor positions.

The LF vibration excitation was provided using the *Modal Shop K2007E01* electromagnetic shaker suspended on strings. The HF ultrasonic excitation was introduced to the beams using low-profile, surface-bonded *Noliac NCE5* monolayer piezoceramic discs (5 mm diameter; 1 mm thickness). The HF actuator was mounted 30 mm from the free end of the beam. Five *Noliac NCE51* piezoceramic discs were used to gather vibro-acoustic responses. Figure 17 presents the geometry of the beam and positions of transducers. Signal generation and data acquisition were handled by the *EC Systems PAQ-G* system. MATLAB was used for signal processing to obtain response spectra and calculate the nonlinear coefficients.

### 4.2. Level of Nonlinearity

**Figure 18:**
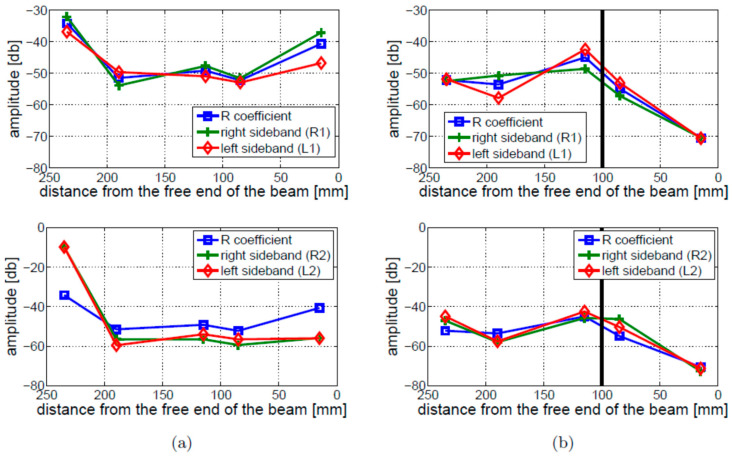
Experimental Test 1—a parametric study of nonlinear vibro-acoustic modulations analyzed using the first (top figures) and second (bottom figures) pairs of sidebands: (**a**) intact beam; (**b**) cracked beam. The crack position is indicated by the vertical black solid line. The horizontal axis corresponds to the measurement locations.

**Figure 19:**
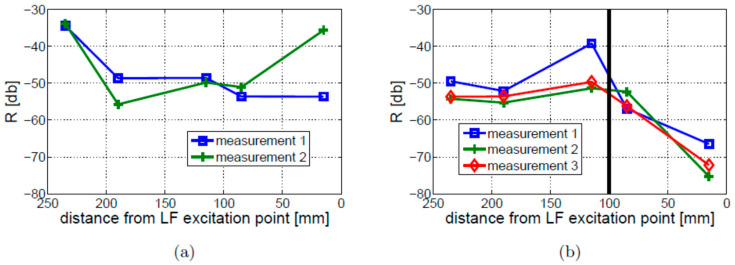
Experimental Test 1—repeatability test for the R coefficient: (**a**) intact beam; (**b**) cracked beam. The crack position is indicated by the vertical solid black line.

Different nonlinear indicators were investigated initially for the intact and crack beams using fixed–free boundary conditions and the Test 1 experimental arrangements from Table 1. Once vibro-acoustic responses were acquired at various positions on the beam, power spectra were calculated and the amplitude of the first two pairs of modulation sidebands was analyzed. The results for the first and second pairs of sidebands are presented in the top and bottom parts of Figure 18, respectively. The results show that relatively small amplitude differences can be observed when the amplitudes of the individual sidebands are compared with the values of the R coefficient. The values of nonlinear indicators are small near the free end of the beam and reach the maxima near the crack position (115 mm away from the free end of the beam) for the damaged beam. The intact beam exhibits the largest values of nonlinear indicators near the fixed end of the beam. The repeatability study was also carried out for the intact and cracked beams to analyze the values of the R coefficient. The results in Figure 19 demonstrate some differences in amplitudes, which are smaller for the cracked beam, but exhibit similar trends for all measurements undertaken. Figure 18 and Figure 19 show that the values of the R coefficient exhibit similar levels for the intact and cracked beams, except for the free end of the beam, where the level of nonlinearity is relatively lower for the cracked beam. Although the nonlinear effect is localized in the cracked beam, crack detection would be difficult in these investigations.

**Figure 20:**
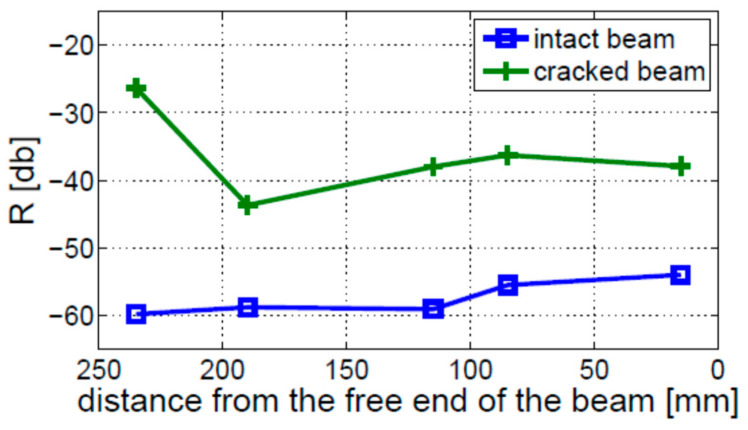
Values of modulation intensity coefficient R measured along the intact and cracked beams (Experimental Test 1) for free–free boundary conditions: (**a**) intact beam; (**b**) cracked beam.

The modulation intensity was also investigated for the free–free boundary conditions. The results are presented in Figure 20. In contrast to the fixed–free boundary conditions, the observed level of nonlinearity for the cracked beam is, in this case, visibly higher than for the intact beam, regardless of sensor location. Moreover, the distribution of nonlinearity along the beam is completely different for free–free and fixed–free boundary conditions. The former does not show any crack localization effect.

### 4.3. Excitation Amplitude

It is known that the level of nonlinearity depends strongly on the amplitude of excitation. Higher excitation amplitudes not only allow the opening–closing action of cracks but also influence the interaction between the structure and boundary conditions. Therefore, the effect of the excitation amplitude was also investigated in this study.

**Figure 21:**
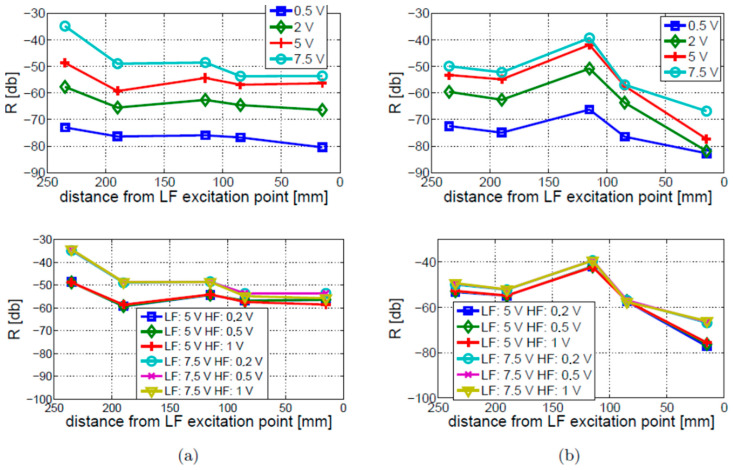
Study of LF vibration (top figures) and HF ultrasonic (bottom figures) excitation amplitude study for (**a**) intact beam; (**b**) cracked beam. Voltage excitation levels are indicated in the legends.

Four amplitude levels of LF vibration excitation (i.e., 0.5, 2, 5, 7.5 V) and three amplitude levels of HF ultrasonic excitations (0.2, 0.5, and 1 V) were considered. The results related to the LF vibration and HF ultrasonic excitations are given in Figure 21. The top part of this figure shows that the modulation intensity is proportional to the amplitude of LF excitation, as expected. This relation holds for both cracked and intact beams. Clearly, a significant part of the measured nonlinearity comes from sources other than damage, which also depend on the amplitude of excitation. Nonetheless, the modulation intensity exhibits local growth around the crack locations for each tested amplitude.

The effect of the HF ultrasonic excitation amplitude is demonstrated in the bottom part of Figure 21. The results demonstrate that the HF ultrasonic excitation amplitude has less effect on the modulation intensity than the LF vibration excitation.

### 4.4. Crack Depth and Location

In this section, we investigate the influence of the crack position and depth on the modulation intensity for the fixed–free and free–free boundary conditions. The highest values of modulation intensity R observed for the intact beam are compared with the lowest values of modulation intensity R observed for the cracked beam in order to assess the worst-case scenario for crack detectability.

**Figure 22:**
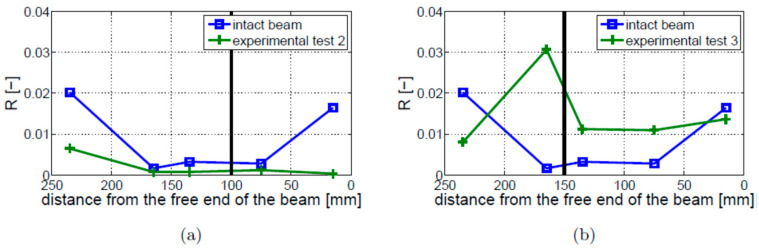
Values of R coefficient for the fixed–free boundary conditions: (**a**) Experimental Test 2; (**b**) Experimental Test 3. Both results are compared with the intact beam results. The crack location is indicated by the vertical solid black line.

The results for the 4 mm crack located at 100 mm (Experimental Test 2) and the 10 mm crack located 150 mm away from the free end of the beam (Experimental Test 3) are shown in Figure 22a,b, respectively. These results are compared with the results for the intact beam. The location of the crack is indicated by a vertical solid black line. The intact beam exhibits the largest value near the ends of the beam. This behavior has not been found for the cracked beams. Interestingly, the level of nonlinearity observed in Experimental Test 2 (Figure 22a) is lower than observed for the intact beam. When Experimental Test 3 is performed (Figure 22b), the values of R are higher only for the measurements taken near the crack (for sensors located 75–165 mm away from the free end of the beam).

**Figure 23:**
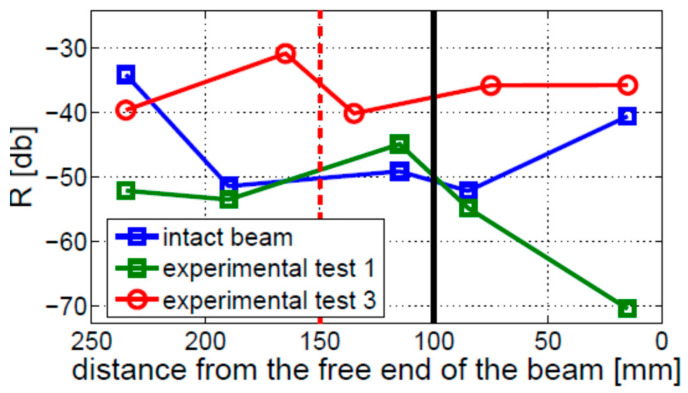
Level of nonlinearity estimated for the fixed–free beam, for two different crack locations indicated by the vertical solid black (Experimental Test 1) and dashed red (Experimental Test 3) lines.

The values of modulation intensity coefficients R estimated for Experimental Tests 1 and 3 are shown in Figure 23. The presented results illustrate the average values from three measurements on the cracked beams and two measurements on the intact beam. The results show that the modulation intensity R observed for the beam in Experimental Test 3 is much higher than that measured on the beam in Experimental Test 1. The difference between the highest and lowest estimated values of the R coefficients in Experimental Test 3 (around 10 dB) is much lower than the relevant difference in Experimental Test 1 (around 25 dB) or for the intact beam (around 20 dB). It is important to note that the crack localization effect is observed in both experimental investigations (i.e., Test 1 and 3). The local growth in modulation intensity was found near the crack location (towards the fixed end of the beam).

**Figure 24:**
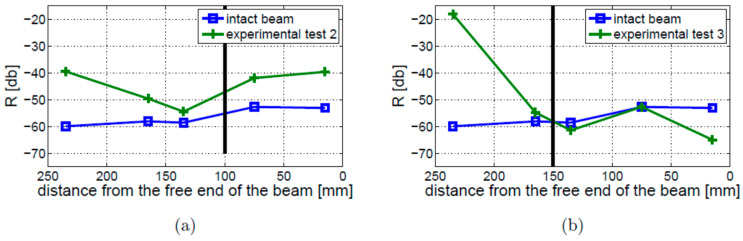
Values of R coefficient for the free–free boundary conditions: (**a**) Experimental Test 2; (**b**) Experimental Test 3. Both results are compared with the intact beam results. The crack location is indicated by the vertical solid black line.

A similar analysis was performed for the free–free boundary conditions. The results of the worst-case scenario measurements—taken for the 4 mm crack (Experimental Test 2) and the 10 mm crack located 150 mm away from the free end of the beam (Experimental Test 3)—are shown in Figure 24a,b, respectively. The results for the intact beam are presented in these figures for comparison. When Experimental Test 2 was performed (Figure 24a), the level of nonlinearity for the cracked beam was always higher than for the intact beam. The crack could be detected but not localized. The results for Experimental Test 3 (Figure 24b) show that the crack can be detected only by the sensor located near the fixed end of the beam.

**Figure 25:**
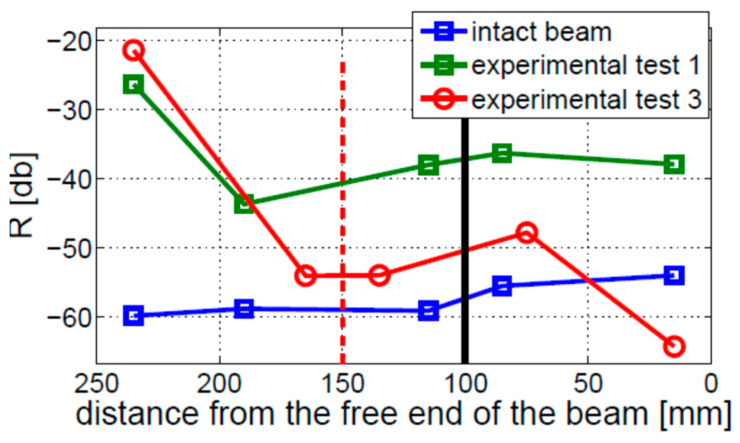
Level of nonlinearity estimated for the free–free beam for two different crack locations indicated by the vertical solid black (Experimental Test 1) and dashed red (Experimental Test 3) lines.

The results for Experimental Test 1 and 3, compared with the results for the intact beam, are given in Figure 25. These results show that the sideband amplitudes estimated in Experimental Test 1 are much higher than those estimated in Experimental Test 3. In neither of the presented experimental tests was the crack localization effect found.

### 4.5. Summary of Experimental Results

The experimental results demonstrate that the level of nonlinearity, estimated by the amplitude of the modulation sidebands, is indeed dependent on the sensor location. The local growth in the nonlinearity is similar to the one found in the simulation studies (Section 3), in which the simplified piezo model of the HF ultrasonic excitation was employed. The most important observations presented in this section can be summarized by the following points:

- The amplitudes of the first two pairs of sidebands are similar. The free–free beam produces similar results compared to the fixed–free beam results in the repeatability tests.
- The values of the R coefficient are proportional to the LF vibration excitation amplitudes for the cracked and intact beams. However, the dependency of the R coefficient on the HF ultrasonic excitation amplitudes has not been found.
- The amplitude of modulation sidebands cannot be used reliably to detect cracks in beams with fixed–free boundary conditions, regardless of the crack size and position. In contrast, cracks can be detected for all investigated crack sizes and locations in beams with free–free boundary conditions.
- The crack localization effect was found in the beam with the fixed–free boundary conditions but not observed in the beams with free–free boundary conditions.

## 5. Conclusions

The nonlinear interaction of longitudinal vibration and ultrasound in beams with cracks was investigated. The major focus was on the localization effect of this interaction, i.e., the locally enhanced nonlinear vibro-acoustic modulation. The work presented involved numerical simulations and experimental tests. The results presented in the paper lead to the following major conclusions.

The crack localization effect was found in numerical simulations and experimental tests for the nonlinear vibro-acoustic interaction. However, the locally induced nonlinearity due to the crack was observed only for cracked beams with fixed–free boundary conditions. This indicates that significant crack perturbation is needed for the effect to occur in practice.

The open crack model used in numerical simulations was unsuitable to reflect the vibro-acoustic interaction properly. The breathing crack model was the best model investigated for the crack localization effect. It is important to note that the crack localization effect was observed in numerical simulations only for the simplified piezo model of HF ultrasonic excitation.

The observation of the crack localization effect in cracked beams—resulting from the nonlinear interaction of longitudinal vibration and ultrasound—is quite significant. This is due to the fact that the longitudinal stiffness of the beam is much higher than the transversal stiffness. The crack perturbations are then significantly lower than for the transversal vibrations of the beam. As a result, the crack localization effect is more difficult to be observed.

Further simulations and experimental work are needed to confirm these findings. Any further work should involve more complex crack–wave interaction models and more experimental tests. The crack localization effect resulting from the interaction of the transversal vibration and ultrasound should also be investigated. Size estimation, along with the localization effect, should also be studied.

## Figures and Tables

**Table 1:** Crack depths and positions investigated in the experimental tests.

Experimental Test	Crack Depth [mm]	Crack Position [mm]
Test 1	10	100
Test 2	4	100
Test 3	10	150

## Data Availability

Data available upon request.
